# HIV positive lactating mothers a missed opportunity in the roll out of the eMTCT strategy in Uganda

**DOI:** 10.1186/1471-2334-14-S2-P24

**Published:** 2014-05-23

**Authors:** Lillian Ayebale, Pamella Bakabulindi, Erasmus Tanga

**Affiliations:** 1 Research Triangle Institute International, Kampala, Uganda

## Introduction

Although Uganda has embraced the strategy of EMTCT by 2015, the strategy focuses mainly on HCT for first ANC visit with less attention on mothers who never attend ANC, deliver in the community and are lactating with unknown status which creates a missed opportunity. The supporting public sector work places Expand Action and Responses to HIV (SPEAR) Project with funding from USAID, has piloted PMTCT/HCT camps/outreaches as an intervention to bridge this gap in their target population.

## Materials and methods

SPEAR supports a given Health Facility to offer outreach services. A two-three days wellness camp that offers HCT, ANC, PNC, FP, Immunization, and general health education is organized in the barracks over a weekend. Prior community mobilization in target barracks that included a film van and door to door mobilization by VHT is carried out.

## Results

559 mothers had HCT during the five camps held at different locations. Of these 103 were pregnant mothers and 456 were lactating mothers. Of the 103, 76 were 1st ANC visits and of the 456 lactating mothers, 306 were receiving 1st HCT since pregnancy and birth of current child. 8 pregnant and 30 lactating mothers were HIV positive.

## Conclusion

Most lactating mothers do not receive HCT for PMTCT and are at a higher risk of transmitting to their babies. About half of the population of pregnant mothers do not attend ANC during pregnancy and deliver in the community unattended by a professional health worker.

## Recommendation

There is need to roll out provider initiated HCT not only at the health facilities, but also at community level for pregnant and lactating mothers in Uganda, for Uganda to achieve virtual EMTCT by 2015.

